# Changes in HCMV immune cell frequency and phenotype are associated with chronic lung allograft dysfunction

**DOI:** 10.3389/fimmu.2023.1143875

**Published:** 2023-04-28

**Authors:** Amélie Rousselière, Laurence Delbos, Aurore Foureau, Martine Reynaud-Gaubert, Antoine Roux, Xavier Demant, Jérôme Le Pavec, Romain Kessler, Jean-François Mornex, Jonathan Messika, Loïc Falque, Aurélie Le Borgne, Véronique Boussaud, Adrien Tissot, Sophie Hombourger, Céline Bressollette-Bodin, Béatrice Charreau

**Affiliations:** ^1^ Nantes Université, CHU Nantes, Inserm, Centre de Recherche Translationnelle en Transplantation et Immunologie, Nantes, France; ^2^ Nantes Université, CHU Nantes, Service de Pneumologie, Institut du thorax, Nantes, France; ^3^ CHU de Marseille, APHM, Hôpital Nord, Service de Pneumologie et Equipe de Transplantation pulmonaire; Marseille, France; Aix-Marseille Université, Marseille, France; ^4^ Hôpital Foch, Service de pneumologie, Suresnes, France; ^5^ Hôpital Haut-Lévêque, Service de pneumologie, CHU de Bordeaux, Bordeaux, France; ^6^ Service de Pneumologie et de Transplantation Pulmonaire, Groupe Hospitalier Marie-Lannelongue -Paris Saint Joseph, Le Plessis-Robinson, France; ^7^ Université Paris-Saclay, Le Kremlin Bicêtre, France; ^8^ UMR_S 999, Université Paris–Sud, Inserm, Groupe hospitalier Marie-Lannelongue-Saint Joseph, Le Plessis-Robinson, France; ^9^ Groupe de transplantation pulmonaire des hôpitaux universitaires de Strasbourg, Inserm-Université de Strasbourg, Strasbourg, France; ^10^ Université de Lyon, Université Lyon1, INRAE, IVPC, Lyon, France; ^11^ Hospices Civils de Lyon, GHE, Service de Pneumologie, Inserm, Lyon, France; ^12^ APHP, Nord-Université Paris Cité, Hôpital Bichat-Claude Bernard, Service de Pneumologie B et Transplantation Pulmonaire, Paris, France; ^13^ Physiopathology and Epidemiology of Respiratory Diseases, UMR1152 INSERM and Université de Paris, Paris, France; ^14^ Service Hospitalier Universitaire Pneumologie et Physiologie, Pôle Thorax et Vaisseaux, CHU Grenoble Alpes, Grenoble, France; ^15^ CHU de Toulouse, Hôpital Larrey, Toulouse, France; ^16^ Service de Pneumologie, Hôpital Européen Georges-Pompidou, Paris, France; ^17^ CHU Nantes, Nantes Université, Laboratoire de Virologie, Nantes, France; ^18^ CHU Nantes, Institut de Transplantation Urologie Néphrologie (ITUN), Nantes, France

**Keywords:** transplantation, CMV, HCMV infection, chronic lung allograft dysfunction, CD8 T cells, HLA-E, UL40, HCMV immunity

## Abstract

**Background:**

Human cytomegalovirus (HCMV) infection is common and often severe in lung transplant recipients (LTRs), and it is a risk factor associated with chronic lung allograft dysfunction (CLAD). The complex interplay between HCMV and allograft rejection is still unclear. Currently, no treatment is available to reverse CLAD after diagnosis, and the identification of reliable biomarkers that can predict the early development of CLAD is needed. This study investigated the HCMV immunity in LTRs who will develop CLAD.

**Methods:**

This study quantified and phenotyped conventional (HLA-A2pp65) and HLA-E-restricted (HLA-EUL40) anti-HCMV CD8^+^ T (CD8 T) cell responses induced by infection in LTRs developing CLAD or maintaining a stable allograft. The homeostasis of immune subsets (B, CD4T, CD8 T, NK, and γδT cells) post-primary infection associated with CLAD was also investigated.

**Results:**

At M18 post-transplantation, HLA-EUL40 CD8 T responses were less frequently found in HCMV^+^ LTRs (21.7%) developing CLAD (CLAD) than in LTRs (55%) keeping a functional graft (STABLE). In contrast, HLA-A2pp65 CD8 T was equally detected in 45% of STABLE and 47.8% of CLAD LTRs. The frequency of HLA-EUL40 and HLA-A2pp65 CD8 T among blood CD8 T cells shows lower median values in CLAD LTRs. Immunophenotype reveals an altered expression profile for HLA-EUL40 CD8 T in CLAD patients with a decreased expression for CD56 and the acquisition of PD-1. In STABLE LTRs, HCMV primary infection causes a decrease in B cells and inflation of CD8 T, CD57^+^/NKG2C^+^ NK, and δ2^−^γδT cells. In CLAD LTRs, the regulation of B, total CD8 T, and δ2^+^γδT cells is maintained, but total NK, CD57^+^/NKG2C^+^ NK, and δ2^−^γδT subsets are markedly reduced, while CD57 is overexpressed across T lymphocytes.

**Conclusions:**

CLAD is associated with significant changes in anti-HCMV immune cell responses. Our findings propose that the presence of dysfunctional HCMV-specific HLA-E-restricted CD8 T cells together with post-infection changes in the immune cell distribution affecting NK and γδT cells defines an early immune signature for CLAD in HCMV^+^ LTRs. Such a signature may be of interest for the monitoring of LTRs and may allow an early stratification of LTRs at risk of CLAD.

## Introduction

Lung transplantation (LTx) has still a poor long-term outcome, with a current median post-transplant survival of 6.5 years (1). Lung transplant recipients (LTRs) have 1-, 5-, and 10-year unadjusted survival rates of 80%, 54%, and 32%, respectively (2). Chronic lung allograft dysfunction (CLAD) is the major cause of failure, accounting for >40% of deaths beyond the first year post-LTx. In most cases, CLAD has an obstructive phenotype known as bronchiolitis obliterans syndrome (BOS) (3, 4). CLAD qualifies the clinical manifestations of a range of pathologic processes in the airway and parenchymal compartments of the lung allograft that lead to a significant and persistent drop in lung function after LTx (5, 6). Allogeneic immune responses and infections are the major triggers of CLAD and allograft failure. Human cytomegalovirus (HCMV) causes the most common infection in LTRs and is an important cause of morbidity and mortality (7, 8). The efficient control of HCMV infection involves both innate and adaptive immune responses (9) and the coordinated action of CD4^+^ T and CD8^+^ αβT (CD8 T), γδT, B, and natural killer (NK) cells (10–13). Consequently, HCMV infection introduces substantial and persistent changes in the homeostasis of immune cells in hosts such as the expansion of the differentiated CD8 T and γδT compartments and the generation of memory-like NK cells expressing NKG2C and CD57 (12, 14–17). Although the mechanisms by which HCMV contributes to CLAD are still unclear, recent findings suggest that the antiviral NK and γδT may play a role (11, 13, 18). In addition to conventional HLA-Ia-restricted CD8 αβT cells directed against a large panel of HCMV peptides (19), HLA-E-restricted CD8 αβT recognizing a limited set of peptides from the viral UL40 protein (HLA-EUL40 CD8 T) has also emerged as frequent and robust CD8 T-cell responses in healthy hosts and transplant recipients (20–24). Nevertheless, how HLA-EUL40 CD8 T could contribute to HCMV control and whether they could trigger allograft rejection are still unclear.

In this study, we investigated the frequency and phenotype of long-lasting HCMV-specific HLA-EUL40 CD8 T-cell responses in comparison with the immunodominant pp65-specific HLA-A2-restricted CD8 T cells (HLA-A2pp65 CD8T) 18 months post-LTx in a cohort of 63 HCMV^+^ LTRs developing CLAD and in matched control LTRs keeping a stable allograft function within 3-5 years post-transplantation. In addition, specific and global HCMV immune cell responses after an HCMV primary infection were further investigated in a longitudinal analysis of blood samples harvested post-infection from LTRs developing a CLAD or keeping a stable allograft. Overall, we identified significant changes in both the frequency and the phenotype of immune responses after HCMV infection which are associated with CLAD.

## Material and methods

### Ethics

The patients’ blood samples and clinical data were collected from the Cohort of Lung Transplantation (COLT) after approval by the institutional ethics board (Comité de Protection des Personnes Ouest 1-Tours, agreement number: 2009-A00036-51). The trial was conducted in accordance with the Declaration of Helsinki. Written informed consent was obtained for all materials from each patient. Patient material was donated voluntarily in accordance with the Declaration of Istanbul.

### Patients and samples

**Figure 1:**
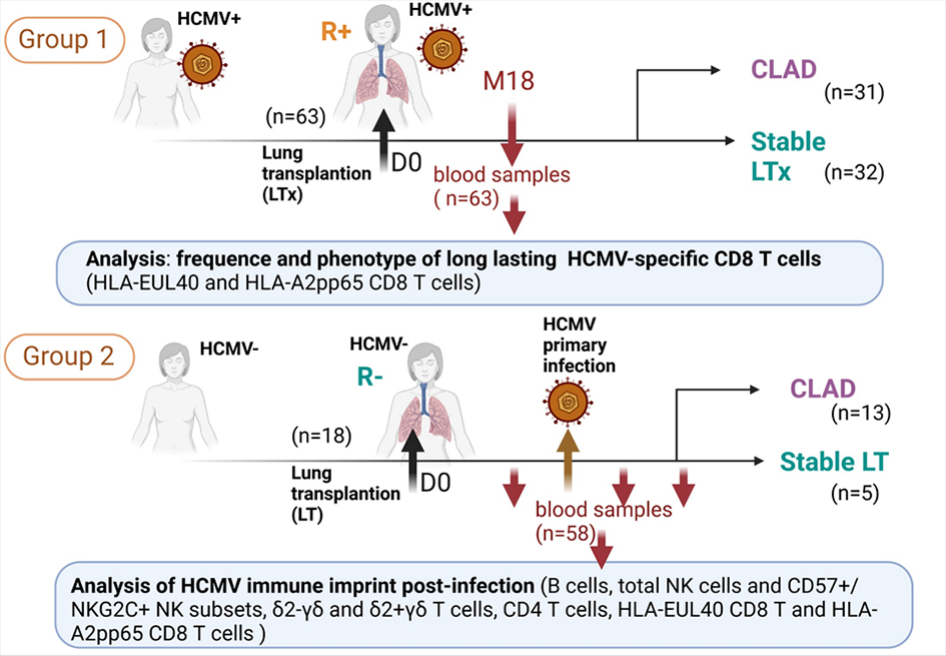
Flowchart representing the design of the study and showing the two groups of lung transplant recipients (LTRs) (group 1 and group 2) included and the samples used for the investigations.

This retrospective study used banked prospectively isolated samples of peripheral blood mononuclear cells (PBMCs) from patients who underwent lung transplantation between 2012 and 2017 and were included in the multicenter longitudinal Cohort of Lung Transplantation (COLT, agreement number: NCT00980967) monitoring patients during 5 years following LTx in order to detect the predictive factors of CLAD. Our study included two groups of LTRs. Group 1 included 63 HCMV seropositive (HCMV^+^, R^+^) before LTx consisting of 31 patients developing a CLAD (referred to as CLAD) and 32 matched control patients without CLAD during the follow-up period of 3 to 5 years (referred to as STABLE). For group 1, a single PBMC sample collected after LTx (around M18 post-LTx) was studied. LTRs were already HCMV seropositive before lung transplantation (R^+^). In this group, only 17 out of the 63 LTRs had an HCMV reactivation episode post-LTx (10/32 STABLES and 7/31 CLAD). Reactivation occurred at 3 to 10 months post-LTx in this group. Thus, the time of virus reactivation was 8 to 15 months before the time of blood collection, and no sample was collected at or close to acute infection. For LTRs with CLAD, samples were analyzed 417 days (mean value from 31 samples) before the CLAD diagnosis. Time from LTx and CLAD diagnosis to sampling for all patients in group 1 are indicated in **Figure S1**. For the longitudinal study, group 2 included 57 PBMC samples from 17 patients with or without CLAD collected before (1 sample/LTR) and after (1-4 samples/LTR) a primary HCMV infection post-transplantation. Sampling according to the time of infection for patients in group 2 is shown in **Figure S2**. A flowchart of the study is provided in **Figure 1**, and the characteristics of the LTRs, groups, and samples are reported in **Table 1**.

**Table 1:** Characteristics of the patients and samples.

	Group 1		Group 2			
	STABLE	CLAD	STABLE	CLAD	*p*-value Group 1	*p*-value Group 2
Lung Tx recipients, *n*	32	31	13	5		
Age [years; median (min-max)]	50 (19-65)	55 (15-64)	28 (18-61)	34 (21-51)	0.0887^a^	0.7560^a^
Gender [female, (%)]	20 (62)	16 (52)	6 (46)	3 (60)	0.4500^b^	>0.9999^b^
Tx donors, *n*	32	31	13	5		
Age [years; median (min-max)]	48 (12-70)	41 (11-68)	39 (14-62)	42 (27-53)	0.2859^c^	0.9414^a^
Gender [female, (%)]	14 (44)	11 (35)	3 (23)	3 (60)	0.6088^c^	0.2682^c^
Tx procedure, *n* (%)					0.1152^d^	>0.9999^b^
Cardiac/lung Tx	1 (3)	1 (3)	0 (0)	0 (0)		
Bilateral lung Tx	26 (81)	18 (58)	12 (92)	5 (100)		
Single lung Tx	5 (16)	12 (39)	1 (8)	0 (0)		
Ischemia [min; median, (min-max)]	252 (1-514)	277 (134-420)	240 (3-335)	320 (205-460)	0.2241^a^	0.2791^a^
CLAD, *n* (%)						
BOS, *n* (%)	NA	17 (55)	NA	5 (100)		
Ras and mixed, *n* (%)	NA	4 (13)	NA	0 (0)		
Median to CLAD diagnosis, post-Tx [months; min-max]	NA	30 (12-69)	NA	31 (24-49)		
Azithromycin	3 (9)	/	7 (54)	/		
HCMV serostatus before LTx						
D^+^/R^+^	18 (56)	21 (68)	0 (0)	0 (0)		
D^−^/R^+^	14 (44)	10 (32)	0 (0)	0 (0)		
D^+^/R^−^	0 (0)	0 (0)	12 (92)	4 (80)		
D^−^/R^−^	0 (0)	0 (0)	1 (8)	1 (20)		
Blood samples, [total], *n*	32	31	41	17		
*Before infection, n*	0	0	13	5		
Median days before infection, (min-max)	NA	NA	182 (103-345)	182 (42-678)	NA	>0.9999^a^
*Post-infection HCMV, n*	32	31	28	12		
Median days post-infection, [min-max]	NA	NA	273.5 (0-746)	407.5 (42-625)		0.0477^a^
Median days post-Tx, [min-max]	561 (506-757)	552 (459-779)	546 (0-1161)	530 (0-1,271)	0.8995^a^	0.7899^a^
Median days to CLAD diagnosis, [min-max]	NA	−257 (−1,576 to 398)	NA	−706 (−1,520 to 395)		

NA, not applicable.

a Mann–Whitney U test.

b Fisher’s exact test.

c t-test.

d Chi-square test.

### HLA-E_UL40_ and HLA-A2_pp65_ tetramer complexes

Peptides from the HCMV UL40 protein (AA_15-23_: VMAPRTLIL, VMAPRTLLL) and the UL83 (pp65) protein (AA_495-503_: NLVPMVATV) were synthesized (purity > 95%) and purchased from GeneCust (Boynes, France). The UL40 peptide sequence from two of the most common variants of the HCMV UL40 protein was selected. The HLA_peptide_ monomers HLA-E*01:01_UL40_ and HLA-A*02:01_pp65_ were designed and produced by the recombinant protein core facilities (P2R, SFR Bonamy, Nantes Université, France) and tetramerized using APC-streptavidin (BD Biosciences, Le Pont de Claix, France) as we previously reported (23).

### Spectral flow cytometry for the detection and quantification of HCMV-specific CD8 T cells, immunophenotyping, and immune subset analyses

Two 13- to 15-color multipanels were designed for a 5-laser spectral flow cytometer (Cytek Aurora™, Cytek Biosciences, Fremont, CA, USA). PBMCs were thawed before immunostaining in RPMI-1640 medium (Gibco, Amarillo, TX, USA) and serially incubated with tetramers and panels of mAbs. Importantly, PBMCs were preincubated with blocking CD94 mAb to avoid non-specific binding of HLA-E tetramers to CD94/NKG2 receptors as we previously described (23–25). A detailed immunostaining protocol and the list of mAbs and reagents used are presented in the Supplementary Material and **Table S1**. The fluorescence intensities were measured using the SpectroFlo™ software version 2.2.0 (Cytek Biosciences). The frequency and phenotype of tetramer-positive (tet+) CD8 T cells and NK cell subpopulations were determined using FlowJo™ Software v10 (BD Biosciences) based on manual gating strategies as previously reported (25) and as shown in **Figure S3**. Immunophenotypes and comparison of immune profiles in samples were also studied using automated optimized parameters for T-distributed stochastic neighbor embedding (opt-SNE), a modified version of t-SNE that enables high-quality embeddings without having to empirically tune algorithm parameters (26), which was done to visualize and to quantify the immunoclusters using the OMIQ software from Dotmatics (www.omiq.ai, www.dotmatics.com).

### Statistical analysis

Data are expressed as means ± SD or represented in box plots or percentages. On each box plot, the central mark indicates the median, the bottom and top edges of the box indicate the interquartile range (IQR), and the whiskers represent the maximum and minimum data points. Appropriate statistical analyses were performed using GraphPad Prism 8.0 Software (GraphPad, San Diego, CA, USA). For the comparison of groups, the Fisher’s exact test, chi-square test, Mann–Whitney *U* test, ANOVA, or two-sided paired *t*-test was used as appropriate. A *p*-value <0.05 was considered to represent a statistically significant difference.

## Results

### The development of CLAD is associated with reduced HCMV-specific HLA-E-restricted CD8 T-cell responses

**Figure 2:**
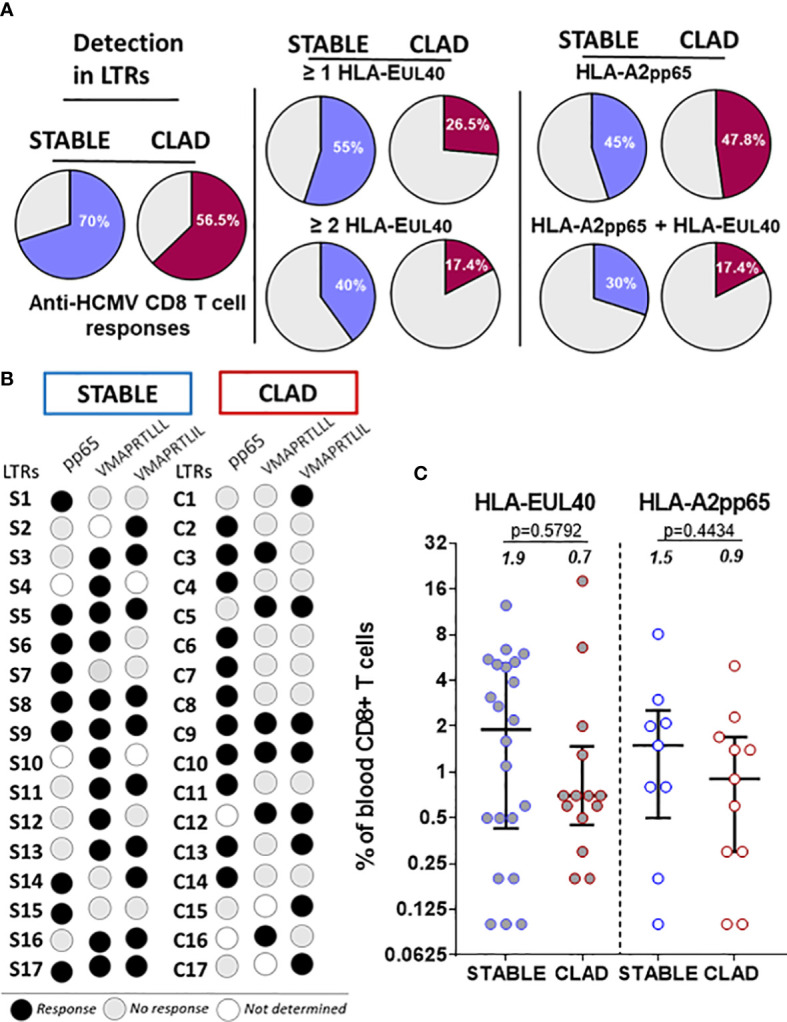
Detection and frequency of HLA-A2pp65 and HLA-E/UL40 human cytomegalovirus (HCMV)-specific CD8 T cells in chronic lung allograft dysfunction (CLAD) *versus* STABLE LTRs. **(A)** Pie charts indicating the percentages of HCMV^+^ LTRs with HCMV-specific CD8 T cells detected at M18 post-transplantation. A single PBMC sample per STABLE (in blue, *n* = 32) and CLAD (in red, *n* = 31) LTRs was used for the concomitant detection of HLA-A2pp65 and HLA-EUL40 CD8 T cells. For the detection of HLA-EUL40 CD8 T cells, two HLA-E tetramers loaded with two different peptides (VMAPRTLLL and VMAPRTLIL) corresponding to the most frequent UL40 variants were used in parallel experiments. The percentages of hosts with at least one type of CD8 T-cell response (A2pp65 or EUL40, left panel) and at least one or two EUL40 CD8 T-cell responses (central panel) with only an A2pp65 response or with both A2pp65 and EUL40 CD8 T-cell responses (right panel) are shown. **(B)** Schematic representation illustrating the patterns of peptide specificities for the anti-HCMV CD8 T cells detected in the STABLE and CLAD HCMV^+^ hosts. In both groups (STABLE and CLAD), 17 out of the 35 LTRs were found to possess at least one HCMV-specific CD8 T response (black dots). LTRs with no CD8 T responses detected are not represented. **(C)** Frequency distribution of HCMV-specific CD8 T cells among the total blood CD8 T cells in STABLE and CLAD LTRs. Scatter plots reporting on the frequency of EUL40 and A2pp65 CD8 T cells in blood samples from LTRs with at least one CD8 T response (STABLE, *n* = 17 LTRs and CLAD, *n* = 17 LTRs). Data are expressed as the percentages of total blood CD8 T cells for each LTR. Medians and IQRs are shown and median values are indicated above the histograms. Each dot corresponds to a single CD8 T-cell subset. Statistical analysis was performed using the Mann–Whitney *U* test.

We first investigated the presence of HCMV-specific CD8 T-cell responses in 63 LTRs consisting of 32 LTRs with a stable allograft and 31 LTRs developing CLAD (group 1, **Table 1**). A single blood sample per recipient, harvested and prospectively banked around M18 post-transplantation, was used to concomitantly detect and quantify immunodominant (HLA-A2pp65) and HLA-E-restricted (HLA-EUL40) CD8 αβT cells in HCMV+ LTRs. Blood samples were collected 593 days (mean values) after transplantation for STABLE (range 506-757 days) and for CLAD (range 459-779 days) LTRs, respectively, as illustrated in **Supplementary Figure S1**. For CLAD LTRs, samples were harvested on average 417 days before the diagnosis of CLAD. HCMV-specific CD8 T cells were detected using HLA-A2pp65 and HLA-EUL40 tetramers and a set of mAbs and quantified after a selective tet+ CD8 T-cell gating as depicted in **Supplementary Figure S3**. Overall, HCMV-specific CD8 T responses were present in 70% of STABLE LTRs but only in 56.5% of CLAD LTRs (**Figure 2A**). Focusing on the specificity of the CD8 T responses, we found that 55% of STABLE had at least one and 40% had at least two HLA-EUL40 CD8 T responses, while 26.5% of CLAD LTRs had at least one and 17.4% had at least two HLA-EUL40 responses. These discrepancies were not observed for the HLA-A2pp65 responses detected equally for 45% of STABLE and 47.8% of CLAD recipients. The concomitant presence of both types of CD8 T cells (HLA-A2pp65 and HLA-EUL40) was found in 30% of STABLE versus 17.4% of CLAD LTRs. The pattern of pp65 and UL40 peptide recognition for LTRs with T-cell responses in both groups is depicted in **Figure 2B**. The frequency of tet+ CD8 T cells, expressed as percentages of total circulating blood CD8 T (**Figure 2C**), varies largely from 0.1% to 18.5% among LTRs and reveals no significant difference between the STABLE and CLAD groups but a trend toward lower median values in CLAD compared with STABLE for both types of responses (HLA-A2pp65 and HLA-EUL40). Thus, in LTRs developing CLAD, there is a possible trend toward a decrease in HCMV-specific CD8 T-cell responses post-infection including a selective decrease in HLA-E-restricted CD8 T-cell responses. However, more evidence is required to establish it definitively.

### HLA-E-restricted HCMV-specific CD8 T-cell responses display an altered phenotype in LTRs developing CLAD

**Figure 3:**
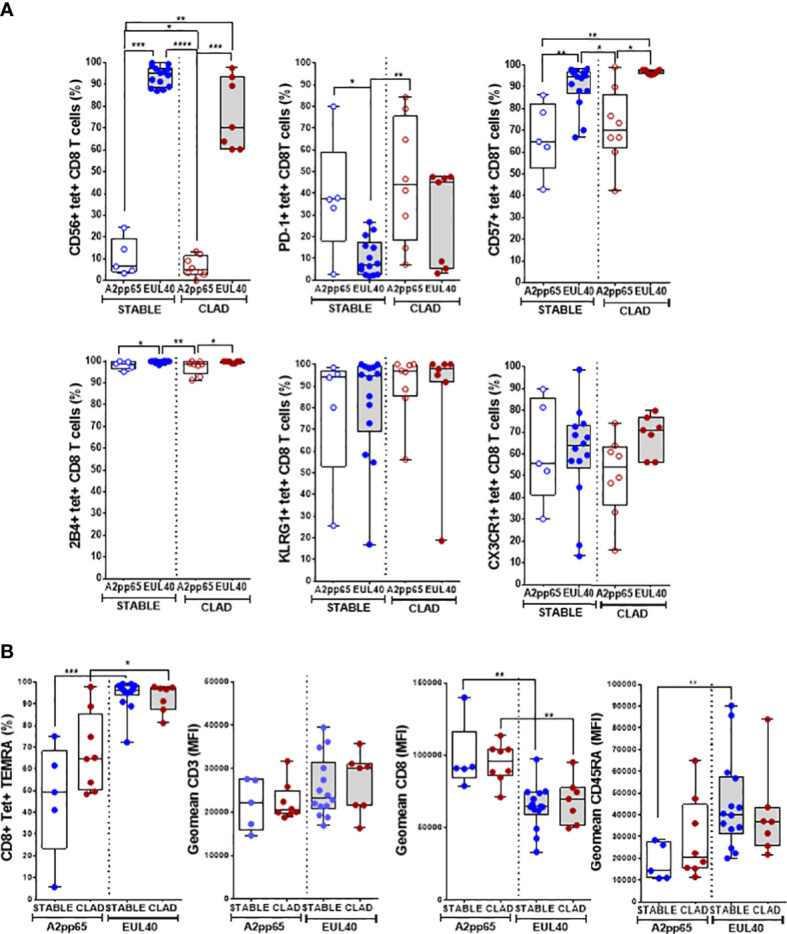
Immunophenotype of HLA-EUL40 and HLA-A2pp65 memory CD8 T cells in CLAD *versus* STABLE LTRs. Immunostaining was performed after a preliminary step of CD94 blocking and by using a panel of mAbs for 11 cell surface markers and HLA tetramer complexes. After fluorescence acquisition, HLA tet^+^ CD8 T populations were selected as CD3^+^CD8^+^TCRγδ^–^ tetramer^+^ T cells and gated as indicated in **Figure S2** to determine the expression of various markers. Analyses are shown in box plots with median and interquartile values. Each dot corresponds to a single, independent tet^+^ CD8 T-cell response including A2pp65 tet^+^ responses from STABLE (*n* = 5) and CLAD (*n* = 8) LTRs and EUL40 from STABLE (*n* = 14) and CLAD (*n* = 7) LTRs. **(A)** Expression of CD56, PD-1, CD57, 2B4, KLRG1, and CX3CR1 on EUL40 and A2pp65 CD8 T cells from STABLE and CLAD LTRs. Results are expressed as percentages of expressing cells among the total tet^+^ CD8 T cells. **(B)** Differentiation stage and expression of TCR co-receptors (CD3, CD8, CD45RA). Percentages of terminally differentiated CD8 T (TEMRA CD45RA^+^/CCR7^−^) cells among the total tet^+^ CD8 T populations. Comparative expression levels for CD3, CD8, and CD45RA on EUL40 and A2pp65 CD8 T cells from STABLE and CLAD LTRs. Data shown are the mean of fluorescence intensity (MFI). **(A, B)** Statistical analysis was performed using the Mann–Whitney *U* test; *p*-values: * for *p* < 0.05, ** for *p* < 0.01, *** for *p* < 0.005, and **** for *p* < 0.001.

**Figure 4:**
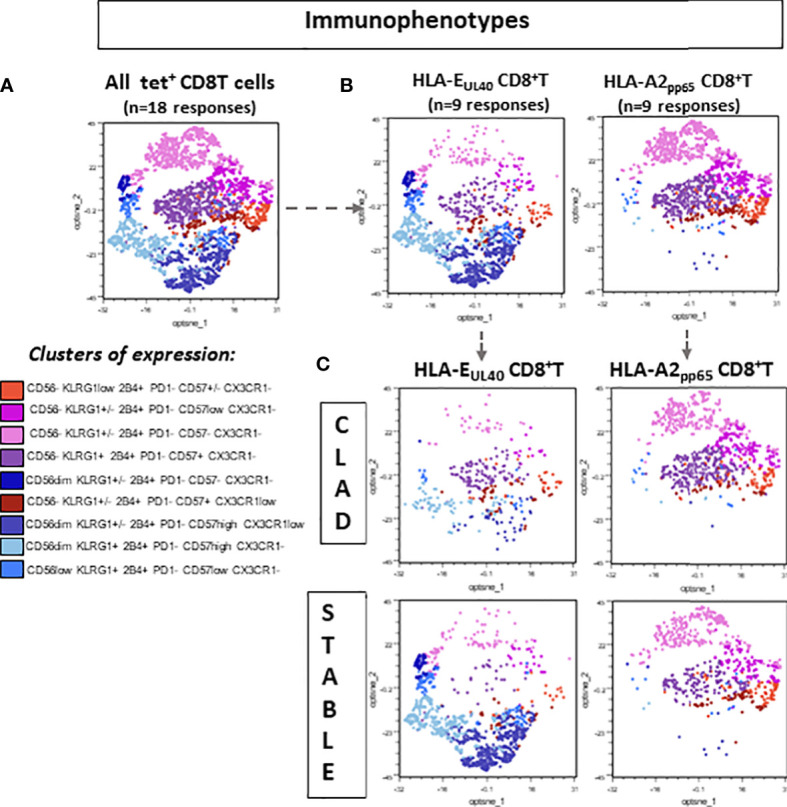
Distinctive signature of CLAD on the immunophenotype of memory EUL40 CD8 T cells. Spectral flow cytometry data were analyzed using a gating strategy depicted in **Supplementary Figure S2** to identify tet^+^ CD8 T cells. Next, tet^+^ CD8 T cells were subgated to select CD3^+^TCRγδ^−^CD8^+^tet^+^ TEMRA (CD45RA^+^CCR7^−^) before clustering analysis. **(A)** 2D opt-SNE visualization of spectral cytometry data showing immunophenotypic patterns in pooled tet^+^ CD8 TEMRA populations (*n* = 18, consisting of nine EUL40 T subsets and nine A2pp65 T subsets). Nine clusters were identified based on the expression of CD57, CD56, CX3CR1, KLRG1, 2B4, and PD-1 as indicated. **(B)** 2D opt-SNE visualization of immunophenotypic patterns in A2pp65 *versus* EUL40 tet^+^ CD8 TEMRA populations (*n* = 9 for EUL40 T subsets and *n* = 9 for A2pp65 T subsets). **(C)** 2D opt-SNE visualization of immunophenotypic patterns in A2pp65 and EUL40 tet^+^ CD8 TEMRA populations from CLAD *versus* STABLE LTRs (*n* = 9 for EUL40 T subsets and *n* = 9 for A2pp65 T subsets).

The T-cell phenotype was investigated to compare the two types of responses (HLA-EUL40 and HLA-A2pp65) in the two groups of LTRs (STABLE and CLAD). **Figure 3A** reports on the expression of CD56, CD57, PD-1, 2B4, KLRG1, and CX3CR1 using manual gating as illustrated in Figure S3. In STABLE LTRs, our data establish CD56, CD57, PD-1, and 2B4 as markers that discriminate HLA-EUL40 from HLA-A2pp65 T cells with HLA-EUL40 displaying a CD56+CD57high2B4highPD-1low phenotype distinct from HLA-A2pp65 CD8 T cells displaying a CD56−2B4+CD57+PD-1+ phenotype. These results are consistent with the immunophenotype that we reported previously for these CD8 T subsets in HCMV^+^ kidney transplant recipients and healthy hosts (23, 24). Comparison of HLA-EUL40 CD8 T in STABLE *versus* CLAD LTRs further reveals a loss of distinctive traits in CLAD LTRs including a decrease in CD56 expression and a trend to a gain in PD-1 expression. In contrast, no changes were found for the HLA-A2pp65 CD8 T subsets. KLRG1 and CX3CR1 were equally expressed by the two CD8 T responses in both groups (STABLE and CLAD). Other characteristics for HLA-EUL40 CD8 T were found in the present study such as being terminally differentiated effector memory CD8 T cells (TEMRA) expressing lower levels of CD8α and higher levels of CD45RA compared with HLA-A2pp65 (**Figure 3B**). These hallmarks were preserved in STABLE and CLAD groups except for CD45RA. To further investigate the phenotype changes associated with CLAD, unsupervised clustering of flow cytometry data was performed on the tetramer-positive (tet+) TEMRA populations from nine LTRs in each group (**Figure 4**). Clustering analysis based on the phenotypic markers CD56, CD57, KLRG1, 2B4, PD-1, and CX3CR1 identified nine clusters of expression that partially segregate HLA-A2pp65 from HLA-EUL40 tet+ CD8 T cells (**Figures 4A, B**). Clusters of cell marker expression defined for HLA-A2pp65 were similar in CLAD and STABLE patients. In contrast, clusters defined for HLA-EUL40 were different in CLAD and STABLE patients. In CLAD LTRs, HLA-EUL40 displays a predominance of clusters typical of HLA-A2pp65 and a loss of clusters specific to HLA-EUL40. The expression of the CD8, CD45RA, CD56, CD57, 2B4, and PD-1 markers in the different clusters for the HLA-A2pp65 and HLA-EUL40 responses and for the STABLE and CLAD LTRs is shown (**Figure S4**). Together, the phenotype analyses associate CLAD with changes in the phenotype of memory HLA-EUL40 CD8 T cells, most probably modifying their functions.

### Changes in HCMV immunity post-infection are associated with the development of CLAD

**Figure 5:**
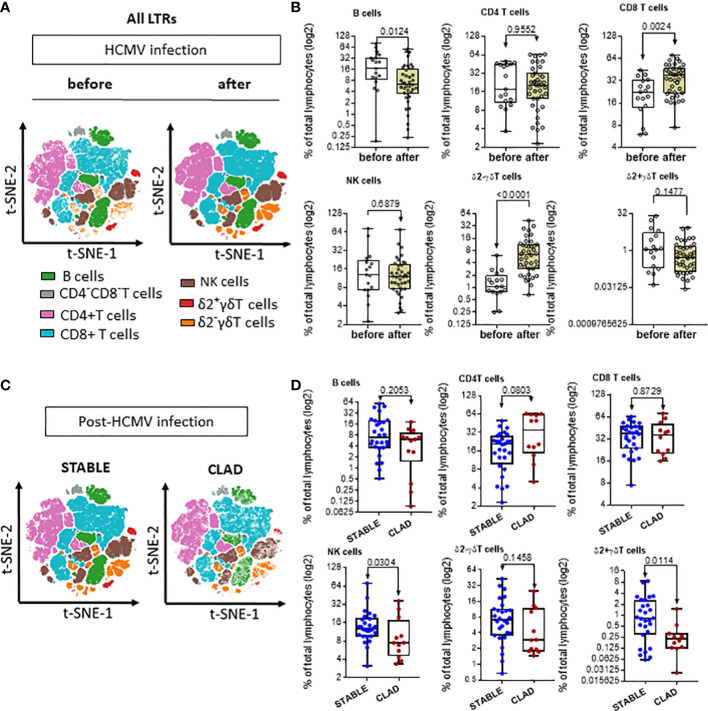
Signature of HCMV infection and signature of CLAD on the distribution of immune subsets in LTRs. **(A)** Visualization (opt-SNE) of the relative abundance of the seven main lymphocyte populations on samples from LTRs (STABLE and CLAD samples embedded) harvested before (*n* = 17) and after (*n* = 40) HCMV infection (left panel). Lymphocyte populations including B, NK, CD4^−^CD8^−^ T, CD4^+^ T, CD8^+^ αβT, δ2^−^γδT, and δ2^+^γδT cells were identified using manual assignation based on phenotype markers (**Figure S5**). **(B)** Frequencies (% total) of the seven main lymphocyte populations identified (right panel). **(C)** Visualization (opt-SNE) of the relative abundance of the seven main lymphocyte populations in samples harvested after HCMV infection from either the STABLE (*n* = 28 samples from 13 LTRs) or CLAD (*n* = 12 samples from 5 LTRs) LTRs (left panel). **(D)** Frequencies (% total) of the seven main lymphocyte populations identified in CLAD *versus* STABLE LTRs (right panel). **(A, B)** Results are shown in whisker plots with median and interquartile values, and each point corresponds to an individual LTR. The groups were compared using the Mann–Whitney *U* test; *p*-values are indicated.

**Figure 6:**
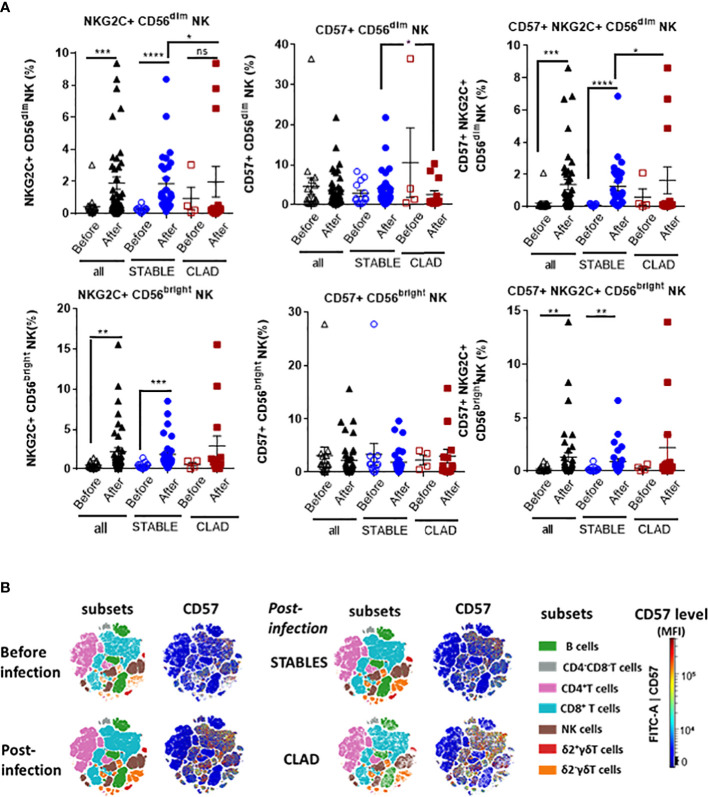
Relative distribution of adaptive/memory NK cell subsets and CD57 level post-HCMV infection in CLAD *versus* STABLE LTRs. **(A)** Spectral flow cytometry data were analyzed to identify NK cell subsets using manual gating. The two main populations of CD3^−^CD16^+^CD56^dim^ and CD3^−^CD16^+^CD56^bright^ NK cells were first segregated and further investigated for the expression and co-expression of the CD57 and NKG2C markers. This analysis included samples harvested before (open labels) and after (closed labels) HCMV infection for all LTRs (*n* = 17 samples before and *n* = 40 samples after the infection, black labels), STABLE LTRs (*n* = 13 samples before and *n* = 28 samples after the infection, blue labels), and CLAD LTRs (*n* = 4 samples before and *n* = 12 samples after the infection, red labels). Results are shown as box blots with median and interquartile values; each point represents a single sample. The groups were compared using the Mann–Whitney *U* test; *p*-values: * for *p* < 0.05, ** for *p* < 0.01, *** for *p* < 0.005, and **** for *p* < 0.001. **(B)** Visualization (opt-SNE) of the relative abundance of the seven main lymphocyte populations on samples and overall CD57 expression level from all LTRs (STABLE and CLAD samples embedded) harvested before (*n* = 17) and after (*n* = 40) HCMV infection (left panel) and (right panel) in samples harvested after HCMV infection from either the STABLE (*n* = 28 samples from 13 LTRs) or CLAD (*n* = 12 samples from 5 LTRs) LTRs (left panel). Lymphocyte populations including B, NK, CD4^−^CD8^−^ T, CD4^+^ T, CD8^+^ αβT, δ2^−^γδT, and δ2^+^γδT cells were identified using manual assignation based on phenotype markers (**Figure S5**).

**Figure 7:**
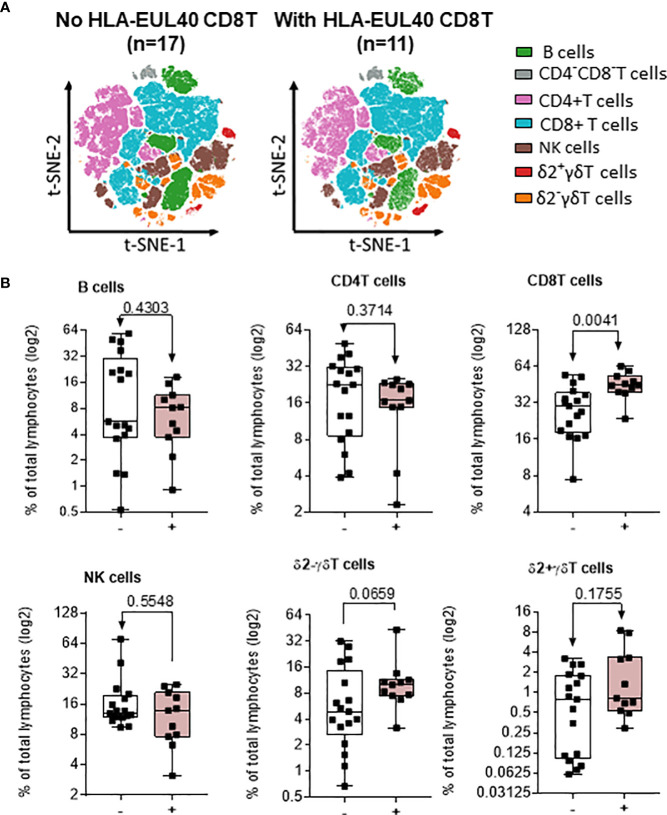
Impact of EUL40 CD8 T cells on the distribution of immune subsets post-infection in STABLE LTRs. **(A)** Visualization (opt-SNE) of the relative abundance of the seven main lymphocyte populations on samples with (*n* = 11) or without (*n* = 17) EUL40 CD8 T detected from STABLE LTRs harvested after HCMV infection. Lymphocyte populations including B, NK, CD4^−^CD8^−^ T, CD4^+^ T, CD8^+^ αβT, δ2^−^γδT, and δ2^+^γδT cells were identified using manual assignation based on phenotype markers. **(B)** Frequencies (% total) of the seven main lymphocyte populations identified in samples harvested after HCMV infection from STABLE LTRs with or without EUL40 CD8 T detected. Results are shown in whisker plots with median and interquartile values, and each point corresponds to an individual sample. The groups were compared using the Mann–Whitney *U* test; *p*-values are indicated.

Next, the global HCMV immune imprint after a primary infection in LTR was deciphered in a group of STABLE and CLAD recipients (group 2, **Table 1**). A panel of mAbs and HLA/peptide tetramers was designed for spectral flow cytometry to identify and quantify, in a single immunoassay, the different lymphocyte subsets including CD3^−^CD19^+^ B cells, CD3^+^CD4^+^ T, CD3^+^CD8 αβT (including tet^+^ HLA-A2pp65 and HLA-EUL40 CD8 T), CD3^+^γδT (including δ2^−^ and δ2^+^γT cells), NKG2C^+^, and/or CD57+CD3−CD56dim and CD3−CD56bright NK cell subsets as we reported previously (25). Unsupervised clustering of cellular markers was used to define cell subsets based on their phenotype (**Figure S5**), and then the frequency of each cell subset was calculated in different conditions. First, immune cell distribution before and after HCMV infection for PBMCs in all samples (embedding STABLE and CLAD LTRs) (**Figures 5A, B**) indicates that the overall imprint of HCMV infection was a significant decrease in the frequency of B cells (~2-fold of the baseline before infection, p = 0.0124) and an increase in the frequency of CD8 T (~2-fold of the baseline before infection, *p* = 0.0024) and δ2^−^γδT (~5-fold of the baseline before infection, p < 0.0001) cells. The analysis of immune subset distribution after HCMV infection was repeated after the segregation of the samples between STABLE and CLAD LTRs (**Figures 5C, D**). In comparison to STABLE, the PBMCs from the CLAD LTRs display decreased frequency in NK cells (~2-fold of the baseline of STABLE, p = 0.0304) and in the δ2+γT (~4-fold of the baseline of STABLE, *p* = 0.0114) subset. Another hallmark of HCMV infection is an elevated frequency in subpopulations of NK expressing NKG2C and/or CD57 in both effector CD56dim and cytokine-producing CD56bright NK subsets (**Figure 6A**). A significant increase was observed in STABLE but not in CLAD LTRs and, thus, may account for the overall decrease in NK cell frequency in CLAD patients. Clustering further revealed an elevated CD57 expression level on NK but also on T cells in LTRs developing CLAD (**Figure 6B**). Finally, the pattern of immune cell post-infection was analyzed in the samples from STABLE LTRs with (n = 11 samples) and without (n = 17 samples) detected HLA-EUL40 CD8 T cells (**Figure 7**). The presence of HLA-EUL40 CD8 T cells in the blood is associated with significant overexpansion of CD8 T cells (~1.5-fold increase, *p* = 0.0041) and a trend toward more δ2^−^γT cells (~2.5-fold increase, *p* = 0.0659). Overall, these results support the idea that, in HCMV^+^ LTRs, changes in the HCMV imprinting post-infection could be an early trigger and/or a signature of CLAD.

## Discussion

Currently, no treatment is available to reverse CLAD after diagnosis. Early identification of CLAD would allow targeted strategies to prevent the progression of injury before irreversible allograft damage occurs. HCMV infection is a risk factor associated with the occurrence of CLAD (27, 28), but the complex interplay between HCMV and allograft rejection is still unclear. The present study investigated HCMV immunity, including both global changes in immune cell homeostasis and peptide-specific CD8 T-cell responses, in LTRs who had an HCMV infection and who will develop CLAD.

Here, we found that the immune signature of a primary HCMV infection in LTRs is associated with a significant increase in total circulating CD8 T, δ2^neg^γδT cell frequency and the emergence of pp65- and UL40-specific CD8 T cells and adaptive NKG2C^+^CD57^+^ NK cells consistent with previous studies (13, 14, 29–31). Our findings report that these changes are paralleled by a decrease in CD19^+^ B-cell rate. A decrease in B cells post-infection was reported in only a few other studies showing that HCMV infection impairs subsequent humoral responses against vaccines due to reduced switched B cells (32) and promotes the expansion of transitional (CD38^hi^CD10^+^) while decreasing memory (CD38^low/neg^CD10^−^IgD^−^CD27^+^) B cells (33). Whether a decrease in total CD19^+^ B cells in HCMV^+^ LTR correlates with quantitative changes in the various B-cell subsets remains to be explored.

When focusing on LTRs who will develop a CLAD, the global HCMV imprint highlighted by the expansion of CD8 T, δ2^neg^γδT cells and the depletion of B cells is conserved. However, the immune profile in CLAD LTRs diverges from the one in STABLE LTRs by displaying reduced percentages in the total circulating NK and adaptive/memory CD57^+^/NKG2C^+^ NK subsets and in δ2^pos^γδT cells. A trend toward more CD4T cells was also observed in CLAD compared with STABLE LTRs (*p* = 0.0803). While NK expansion correlates with efficient control of HCMV infection (34), recent studies indicated that the antiviral NK cell response to HCMV may play a role in CLAD (35, 36). In parallel to HLA-EUL40 CD8 T, adaptive memory NKG2C^+^ NK cells proliferate in response to HCMV infection *via* the presentation of virally encoded UL40 peptides on HLA-E molecules. It has been reported that defects in blood NK cells in CLAD may be due to the sequestration of these cells in lung allografts (11). NK cells may mediate opposite and still debated effects on lung transplant outcomes (37). A likely mechanism to account for the NK cell-mediated alloreactivity is based on the “missing self,” whereby NK cells can recognize and specifically kill cells that have downregulated, such as infected cells, or mismatched MHC class Ia molecules such as allograft vascular cells (38). NK cells may also contribute to transplant rejection by promoting allograft injury through direct cell lysis by ADCC (39). Moreover, NK cells might also promote graft tolerance through the elimination of the graft’s antigen-presenting cells subsequently reducing alloreactive CD4^+^ T cells (40). It can be observed that our study cohort was not large enough for patient stratification according to parameters related to either infection, CLAD phenotype, previous rejection episode, or immune status. This needs to be addressed in future studies.

The presence of HLA-EUL40 CD8 T cells in the blood of LTRs has been previously shown to correlate with CLAD but was examined in only a few LTRs (22). Our study indicates that the occurrence of CLAD in HCMV^+^ recipients is associated with a lower frequency in UL40-specific HLA-E-restricted CD8 T among LTRs as well as with the loss of their distinctive phenotype. Whether the drop in circulating HLA-EUL40 CD8 T cells could be due to their presence in the graft cannot be excluded, but this was not investigated in the present study. Moreover, we cannot exclude that HCMV-specific CD8 T cells representing less than 0.1% of the total CD8 T pool (our threshold of detection) were also present in this cohort but were not detected in our experiments. We cannot exclude the possibility that CD8 T cells targeting other UL40 variants were also present in this cohort but were not detected using our protocol. Consistent with our previous studies on HCMV^+^ kidney transplant recipients (23, 24), here, we show that HLA-E-restricted CD8 T cells display a distinctive phenotype featured by the expression of CD57, CD56, and CD45RA and a low level of CD8 and PD-1 in HCMV^+^ lung transplanted hosts. Nevertheless, our results reveal that the phenotype of HLA-EUL40 CD8 T may also be modulated as highlighted in our study by the partial loss of CD56 expression and gain of PD-1 in CLAD LTRs which bring HLA-EUL40 CD8 T phenotypically closer to HLA-A2pp65 CD8 T. The potential impact of a previous rejection episode or virus reactivation on the cell phenotype was not investigated here. We have no data supporting this hypothesis, and no statistical analysis was possible due to the small size of the cohort. Furthermore, our previous studies investigating the phenotype of HLA-EUL40 CD8 T cells in various groups of hosts (HCMV seropositive healthy individuals and kidney transplant recipients) following primary infection or reactivation post-transplantation (23, 24, 41) indicated that the phenotype and, in particular, PD-1 and CD56 levels were stable.

While the causes and the consequences of these changes still remain to be studied, this is, to our knowledge, the first evidence for the plasticity of the HLA-EUL40 CD8 T phenotype. During acute antigen exposure, it has been shown that antigen-specific CD8 T cells that survive to the memory stage acquire a methylated PD-1 promoter, whereas during the persistent antigen exposure of a chronic infection such as HCMV antigens, the antigen-specific CD8 T cells retain a demethylated PD-1 locus allowing the expression of PD-1 (42). Distinct expression for PD-1 on HLA-EUL40 compared with HLA-A2pp65 CD8 T may suggest that such epigenetic regulation occurs in HLA-A2pp65 but not in HLA-EUL40 CD8 T cells except in conditions promoting CLAD in LTRs. It can thus be speculated that, in developing CLAD, HLA-E-restricted CD8 T cells acquire the phenotype and most probably the functions of HLA-A2-restricted CD8 T cells. The acquisition of the inhibitory receptor PD-1 provides a way to negatively regulate HLA-EUL40 CD8 T-cell function and could also be indicative of T-cell exhaustion or senescence (43). In addition, the loss of CD56 is another hallmark of HLA-EUL40 CD8 T cells in developing CLAD which may reflect a decline in their cytotoxic capacity since CD56 is associated with potent cytolytic functions in NK and T cells (44, 45). Thus, a compromised profile of HLA-EUL40 CD8 T cells may be the consequence of an immune and inflammatory environment associated with CLAD development. Functionally, the compromised phenotype for HLA-EUL40 CD8 T and the reduced adaptive NK cell rate may also cause a less effective control of HCMV infection promoting graft injury and CLAD. HMCV-infected, HLA-E-expressing cells including HCMV targets such as hematopoietic and endothelial cells (46) may thus escape from effector memory T and NK cell-mediated lysis. In STABLE LTRs, the detection of HLA-EUL40 CD8 T is associated with a significantly higher expansion of total CD8 T and δ2^−^γδT cells, thus most probably reflecting an efficient and protective HCMV immunity.

The management of lung recipients identified as having a risk of developing CLAD could allow personalized healthcare to improve quality of life. For such risk stratification, reliable biomarkers that can predict the early development of CLAD are needed. In this setting, our findings further propose that the detection of dysfunctional EUL40 CD8 T together with changes in the global immune cell distribution affecting NK and γδT cells defines an early immune signature for CLAD in HCMV^+^ LTRs. Such a signature may be of interest for the monitoring of LTRs and may allow an early stratification of LTRs at risk of CLAD.

## Data availability statement

The raw data supporting the conclusions of this article will be made available by the authors, without undue reservation.

## Ethics statement

The studies involving human participants were reviewed and approved by Comité de Protection des Personnes Ouest 1-Tours, France, agreement number: 2009-A00036-51. The patients/participants provided their written informed consent to participate in this study.

## Author contributions

Concept and design: AmR, LD and BC. Acquisition, analysis, orinterpretation of data: AmR, BC, SH, LD, AF, CB-B and AT. Draftingof the manuscript: AmR and BC. Statistical analysis: AmR, SH and BC.Administrative, technical, funding, or material support: CB-B, AT,AF, MR-G, AnR, XD, JL, RK, J-FM, JM, LF, AL and VB. All authorscontributed to the article and approved the submitted version.
