# Supplementary material for: Changes in HCMV immune cell frequency and phenotype are associated with chronic lung allograft dysfunction
Source: Front Immunol. 2023 Apr 28;14:1143875. doi: 10.3389/fimmu.2023.1143875 (PMC10175754; doi:10.3389/fimmu.2023.1143875)
Supplement: Supplementary file 1 [file DataSheet_1.pdf]

SUPPLEMENTARY DATA

Immunostaining protocol for Spectral flow Cytometry

Immunostaining protocols mixing antibodies and HLApeptide tetramer complexes were set up for use on a Cytek Aurora (Cytek Biosciences, Fremont, CA, USA) spectral flow cytometry platform with a 5-laser configuration (laser excitation wavelenghts: 355nm, 405 nm, 488 nm, 561 nm, et 640 nm). Before use, titration experiments were carried out to determine the mAb concentration providing highest staining index. Firstly, for detection, quantification and immunophenotyping, of HCMV-specific CD8T cells PBMC (1.10^6 PBMC/well in 96-well plates) were costained using a multistep protocol. PBMC are washed twice in RPMI and cells and filtered through a 100 µm filter (ThermoFisher, Waltham, MA USA), before immunostaining. PBMC were then incubated first with a viability marker, Fixable Viability Stain 440UV (BD Bioscience), diluted in PBS for 15 min at 4°C. PBMC were washed twice in PBS and centrifugated at 2500 rpm for 2 min at 4°C. Next, cells were incubated with anti-CD94 mAb (BD Bioscience) to avoid the binding of HLA-E_{UL40} tetramer to CD94/NKG2A/C receptors. After a washing step, PBMC were incubated with APC-labeled -$HLA_{peptide}$ tetramers (50µg/mL in PBS), for 20 min at RT. For detection, quantification and immunophenotyping, of HCMV-specific CD8T cells PBMCs were then incubated successively for 10 min at 4°C with cocktails of antibodies diluted in PBS: containing Fc-block™ reagent (BD Bioscience) and specific mAbs against CD3, CD8, CD45RA, CCR7, CD56, CD57, 2B4, KLRG1, PD-1, -CCR7, TCRγδ and CX3CR1. For global immune subset analysis PBMC were incubated with anti-NKG2A and NKG2C prior the CD94 blocking step. After a washing step, PBMC were incubated with APC-labeled -$HLA_{peptide}$ tetramers (50µg/mL in PBS), for 20 min at RT. Cells were then incubated successively for 10 min at 4°C with cocktails of antibodies diluted in PBS: containing Fc-block™ reagent (BD Bioscience) and specific mAbs against CD3, CD4, CD8, CD16, CD45RA, CD56, CD57, TCRγδ , TCRγδ2, and CD158. After a washing step, PBMC are finally incubated with anti-CD19 mAbs for 15 min at 4°C. All antibodies are listed in **Table S1**. PBMC are washed twice and were resuspended in PBS before fluorescence analysis. The fluorescence intensities were measured with a five-laser Cytek Aurora™ spectral flow cytometer (Cytek Biosciences) using SpectroFlo™ software version 2.2.0 (Cytek Biosciences). By using online fluorescence spectra viewers, we were able to identify 20 fluorophores with distinct signatures that could be

used in the panel. The selected fluorophores include BUV395, UV440, BUV496, BUV563, BUV737, BUV805, BV421, VioBlue, BV510, BV570, BV605, BV785, FITC, PerCPeFluor710, PE, AlexaFluor594, PE-Cy7, SparkNir685, APC Fire750 and APC (**Table S1**). The spectral profile of unstained cells was collected and treated as an independent parameter, which allows the autofluorescence signature to be extracted using the unmixing algorithm. The full emission spectrum of each single-stained sample was performed using compensation beads (OneComp eBeads™ , Thermo Fisher) or PBMC and was used to determine the contribution of each fluorophore in a mixed sample using spectral deconvolution (unmixing) algorithms before experiments. The fluorophore spectral signatures obtained at the cytometer were compared to the gold standard « Full Spectrum Signatures » shown in the Aurora Fluorochrome Guide (https://cytekbio.com/blogs/resources/5l-full-spectrum-cytometry-overview-poster) to ensure fluorophore identity and quality. Post-acquisition, unmixed FCS files were conventionally compensated before the data analysis. The frequency of major immune cell populations was determined using FlowJo™ Software v10 (BD Biosciences) based on manual gating strategies as reported on the results section.

Table S1. Antibodies and fluorochromes used in multipanel spectral flow cytometry for the analyses of PBMC samples from Group 1 and Group 2 LTRs.

Excitation λ (nm)	Fluorochrome	Emission λ (nm)	Cytometer Detector	Antigen	Antibody Clone	Source
355	BUV395	395	UV2	CD45RA	5H9	BD Bioscience
	Fixable Viability Dye / UV440	436	UV6	necrotic cells	/	BD Bioscience
	BUV737	735	UV14	CD56	NCAM16.2	BD Bioscience
	BUV805	803	UV16	CD8	SK1	BD Bioscience
405	BV421	421	V1	CCR7	G043H7	Biolegend
	Vioblue	452	V3	KLRG1	REA261	Myltenyi
	BV510	510	V7	CD3	OKT3	Biolegend
	BV605	603	V10	2B4	C1.7	Biolegend
	BV785	785	V15	PD1	EH12.2H7	Biolegend
488	FITC	520	B2	CD57	HNK-1	Biolegend
	PerCPeFluor710	710	B10	TCRgd	B1.1	ThermoFisher
561	PE-Cy7	781	YG9	CX3CR1	2A9-1	Biolegend
640	APC	660	R1	Streptavidin-HLA/peptide	/	BD Bioscience
NA	Blocking mAb	NA	NA	CD94	HP-3D9	BD Bioscience
NA	/	NA	NA	Fc Block	Fc1.3216	BD Bioscience

Excitation λ (nm)	Fluorochrome	Emission λ(nm)	ytometer Detector	Antigen	Antibody Clone	Source
355	Fixable iability Dye / UV4	436	UV6	Necrotic cells	/	BD Bioscience
	BUV496	496	UV7	CD16	3G8	BD Bioscience
	BUV563	564	UV9	NKG2C	134591	BD Bioscience
	BUV737	735	UV14	CD56	NCAM16.2	BD Bioscience
	BUV805	803	UV16	CD8	SK1	BD Bioscience
405	BV510	510	V7	CD3	OKT3	Biolegend
	BV570	570	V8	CD4	RPA-T4	Biolegend
488	FITC	520	B2	CD57	HNK-1	Biolegend
	erCPeFluor710	710	B10	TCRgd	B1.1	ThermoFisher
561	PE	576	YG1	CD158	HP-MA4	Biolegend
	lexaFluor594	617	YG3	NKG2A	131411	Bio-Techne
	Pe-Cy7	781	YG9	HLA-E	3D12	Biolegend
640	SparkNIR685	685	R3	CD19	HIB19	Biolegend
	APC-Fire750	787	R7	TCRgd2	B6	Biolegend
	APC	660	R1	reptavidin- _A/peptide	/	BD Bioscience
NA	Blocking mAb	NA	NA	CD94	HP-3D9	BD Bioscience
NA	/	NA	NA	Fc Block	Fc1.3216	BD Bioscience

Figure S1

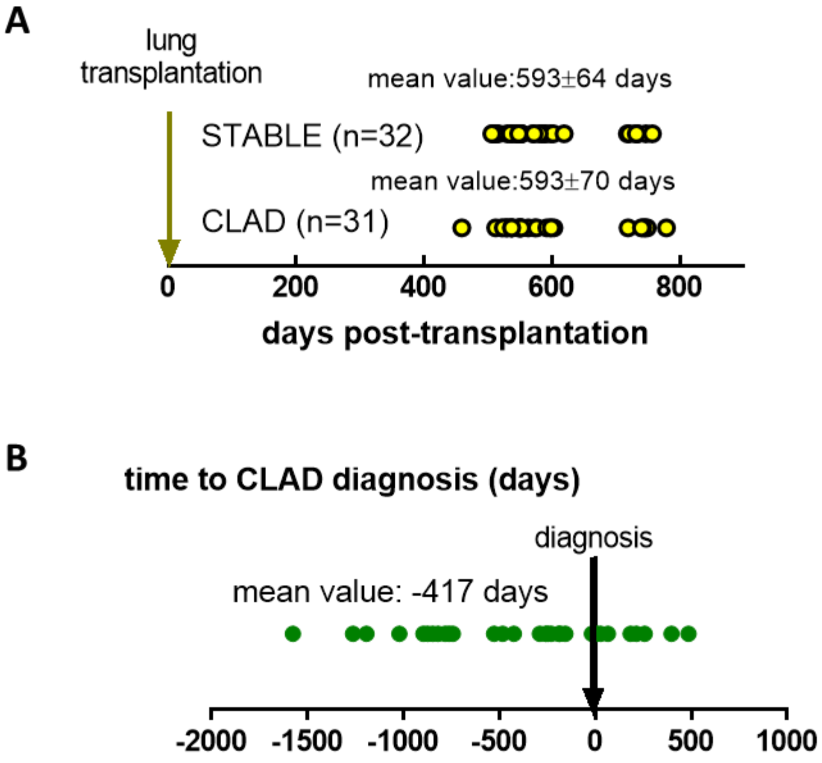

Figure S1. Schematic representation of blood samples from Group1's LTR for STABLE (**A**) and CLAD patients and their distribution toward lung transplantation (arrow), means values and SD are indicated. (**B**) Samples from CLAD LTR according to the time of CLAD diagnosis (arrow), mean value is indicated**.**

Figure S2

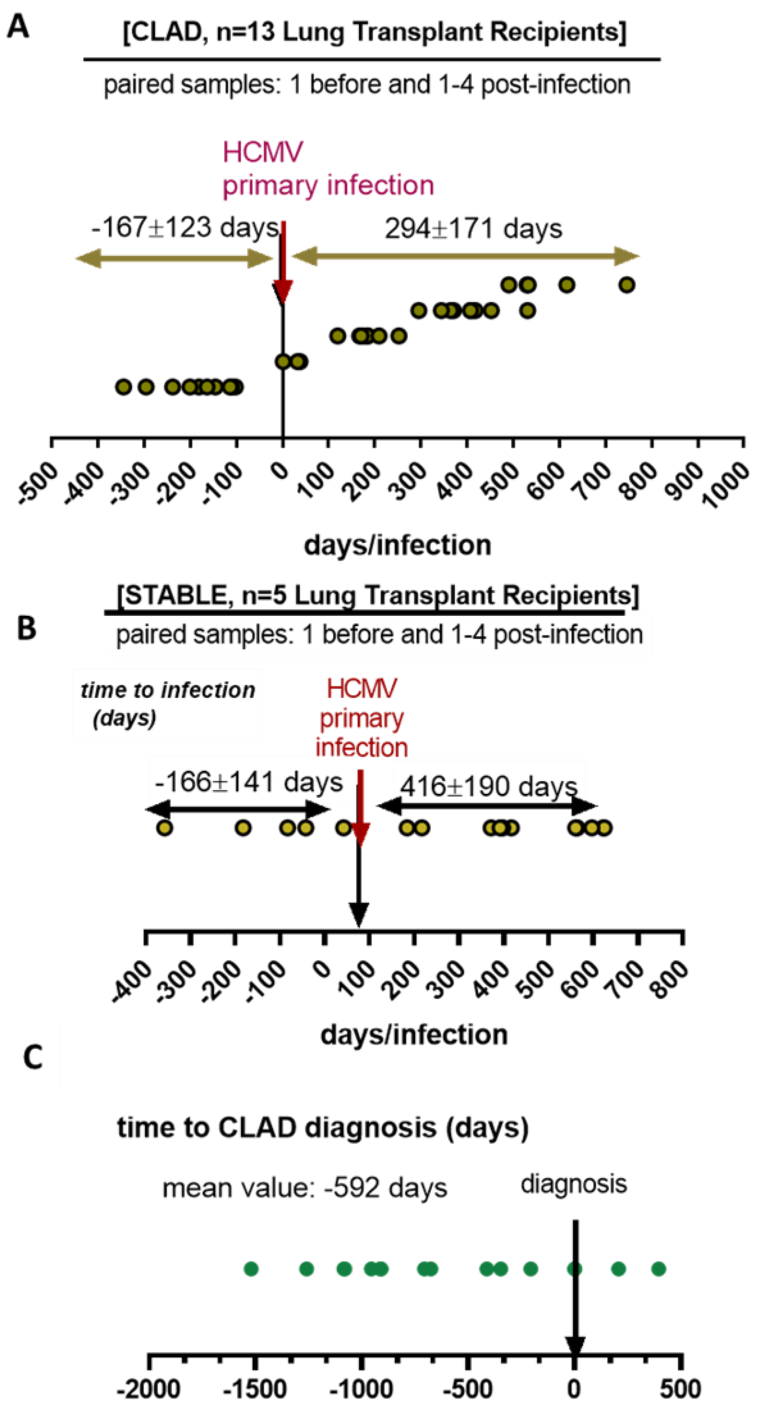

Figure S2. Schematic representation of blood samples from Group2's LTR for STABLE (**A**) and CLAD (**B**) patients and their distribution toward HCMV primary infection (arrow), means values and SD are indicated. (**C**) Sampling from CLAD LTR according to the time of CLAD diagnosis (arrow), mean value is indicated**.**

Figure S3

A

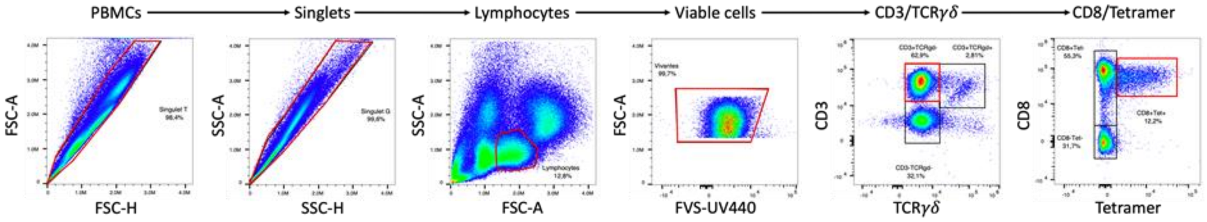

B

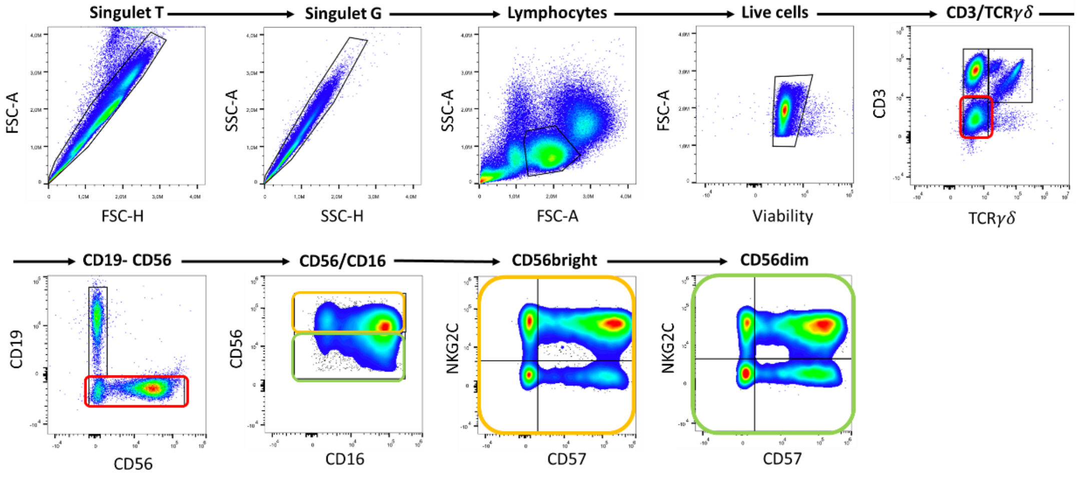

Figure S3. Gating strategies used for the analyses of flow cytometry data for (A) the quantification and phenotyping of HLA-EUL40 and HLA-A2pp65 tetramer positive CD8 T cells and **(B)** the analysis of NK cell subsets using costaining for CD56, CD16, NKG2C and CD57 antigens.

Figure S4

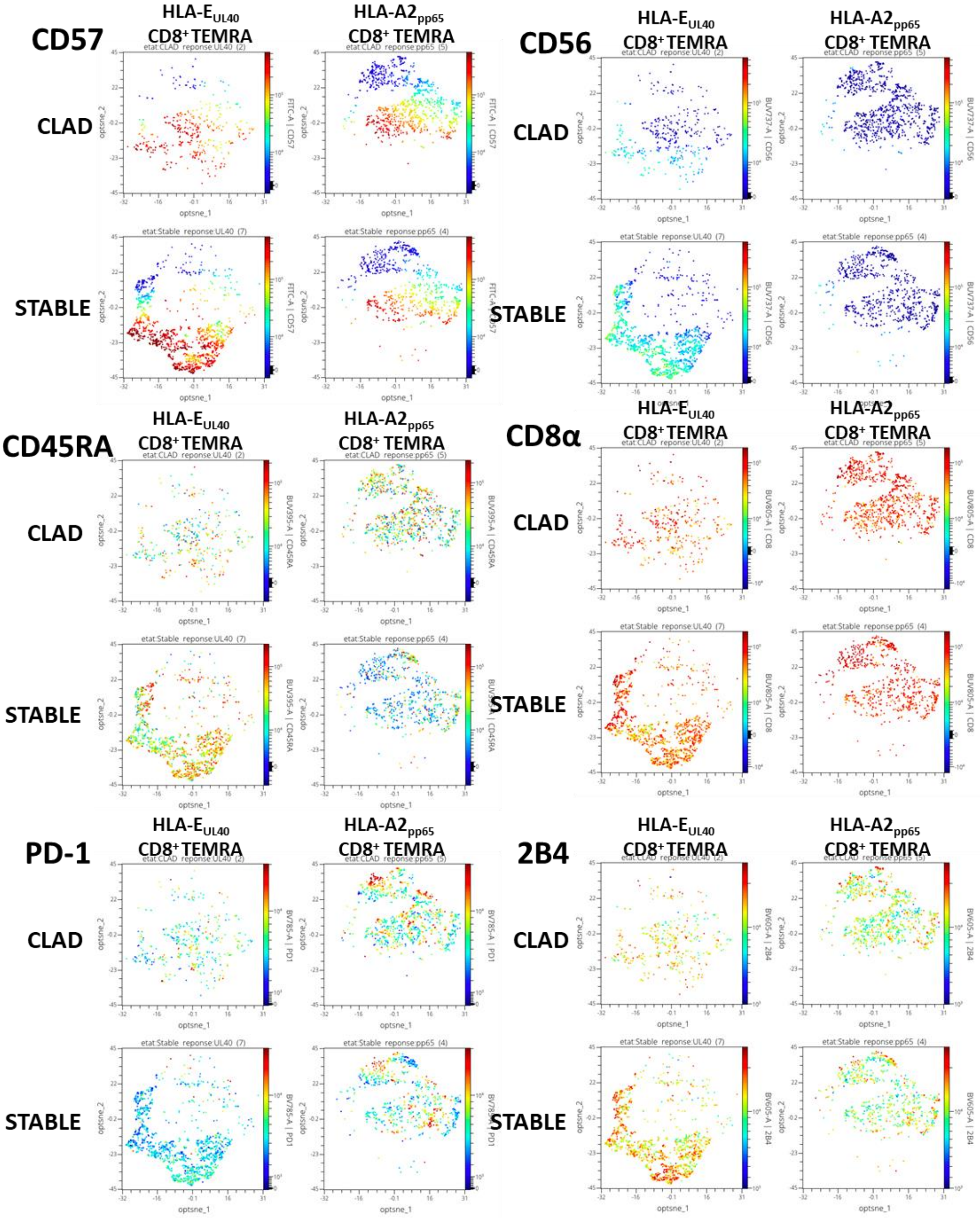

Figure S4. 2D t-SNE analyses showing the expression of the CD8, CD45RA, CD56, CD57, 2B4 and PD-1 markers in the different clusters identified by OMIQ for the HLA-A2pp65 and HLA-EUL40 CD8T responses in STABLE and CLAD LTR.

Figure S5

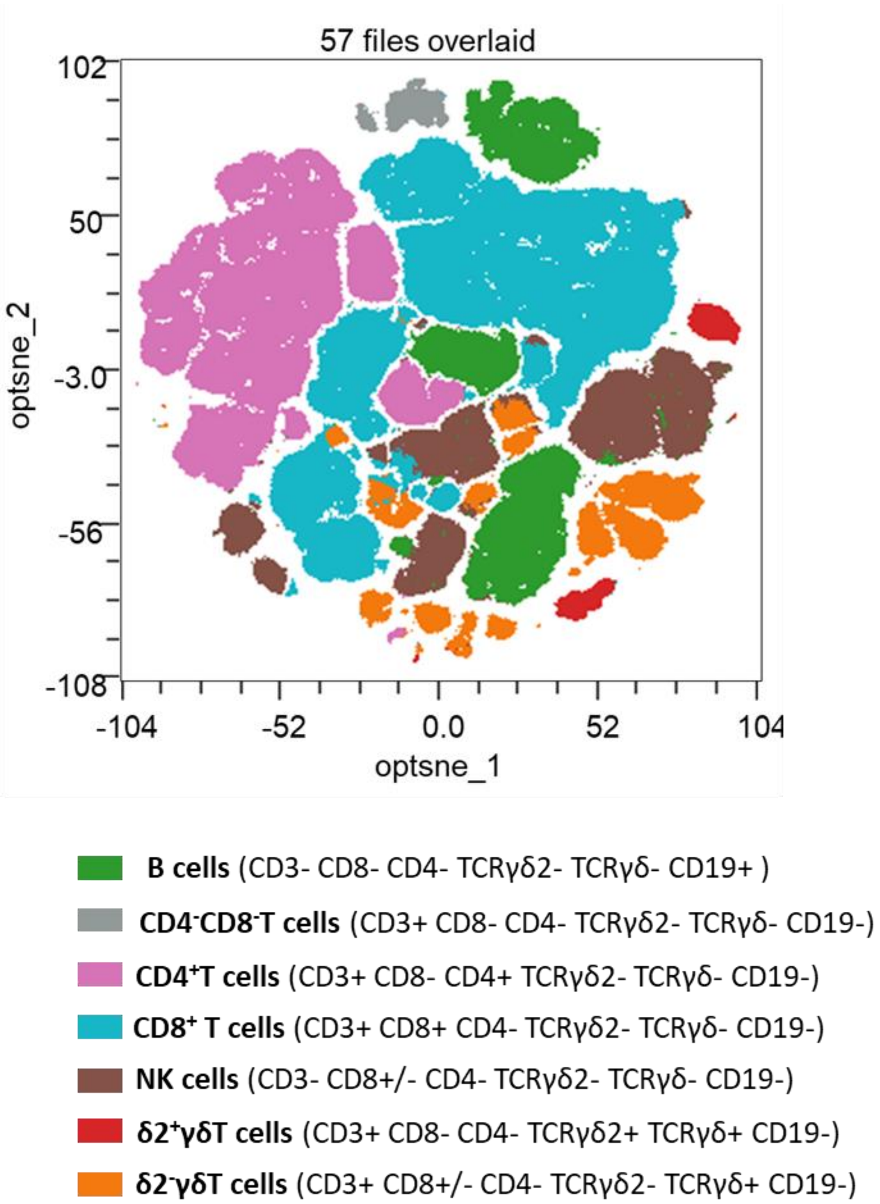

Figure S5. Immune cell assignation and clustering. 2D opt-SNE visualization of spectral cytometry data from 57 PBMC samples**.** Immune subsets were divided into seven clusters defined according to their immunophenotypes. Cell markers used for the assignation of the clusters are indicated in the caption**.**
