# Supplementary figures and images for: Changes in HCMV immune cell frequency and phenotype are associated with chronic lung allograft dysfunction
Source: Front Immunol. 2023 Apr 28;14:1143875. doi: 10.3389/fimmu.2023.1143875 (PMC10175754; doi:10.3389/fimmu.2023.1143875)

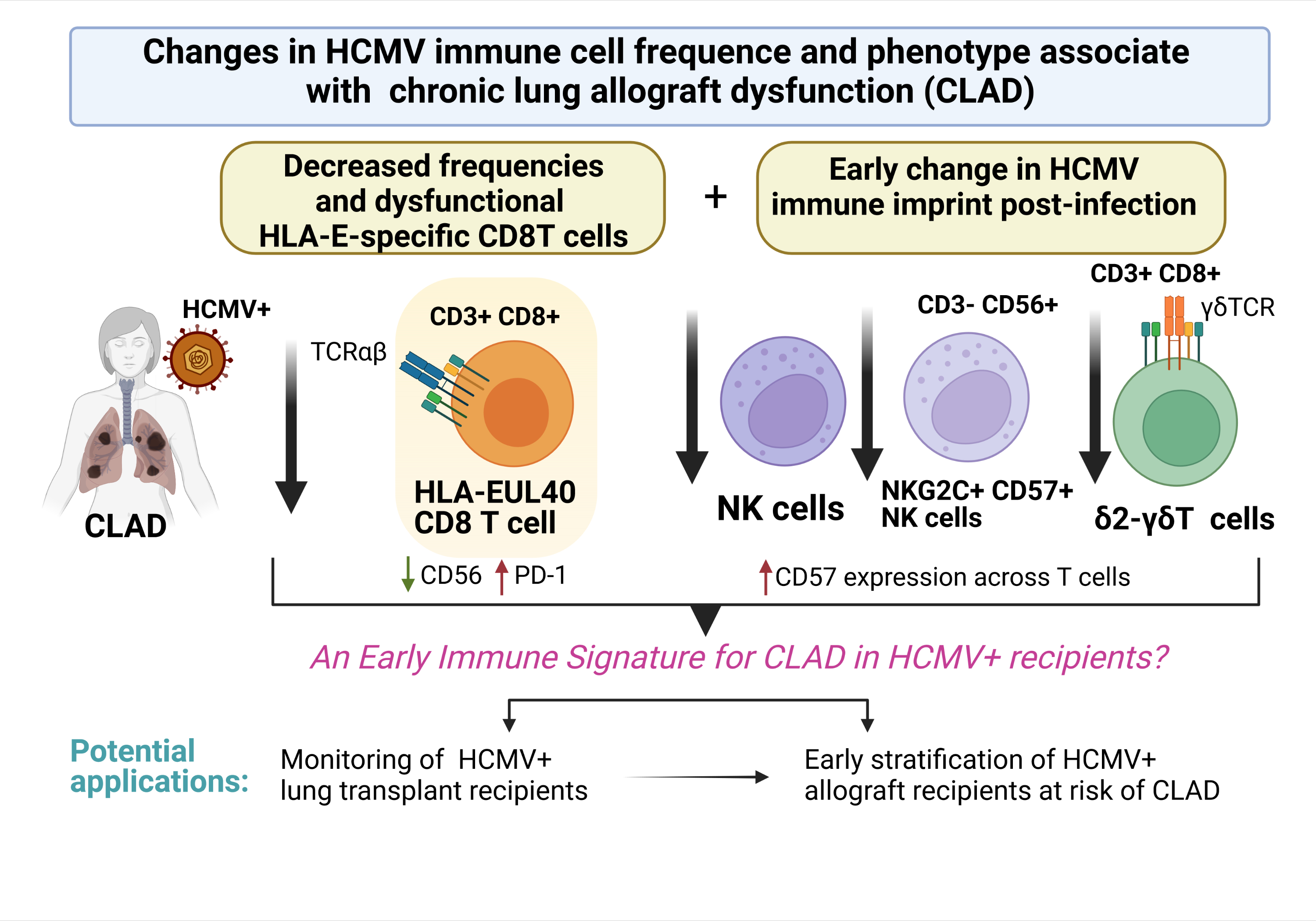

Supplement: Supplementary file 2 [file Image_1.jpeg]
